# Comparing the diagnostic performance of radiotracers in recurrent prostate cancer: a systematic review and network meta-analysis

**DOI:** 10.1007/s00259-021-05210-9

**Published:** 2021-02-06

**Authors:** Ian Leigh Alberts, Svenja Elizabeth Seide, Clemens Mingels, Karl Peter Bohn, Kuangyu Shi, Helle D. Zacho, Axel Rominger, Ali Afshar-Oromieh

**Affiliations:** 1 Department of Nuclear Medicine. Inselspital, Bern University Hospital, University of Bern, Street: Freiburgstr. 18, CH-3010 Bern, Switzerland; 2 Institute of Medical Biometry and Informatics, University of Heidelberg, Im Neuenheimer Feld 130.3, 69120 Heidelberg, Germany; 3 Department of Nuclear Medicine and Clinical Cancer Research Center, Aalborg University Hospital, Hobrovej 18-22, DK-9000 Aalborg, Denmark

**Keywords:** Network meta-analysis, PET/CT, Positron emission tomography, PSMA, Choline, Radiotracers, Comparative imaging

## Abstract

**Purpose:**

Many radiotracers are currently available for the detection of recurrent prostate cancer (rPC), yet many have not been compared head-to-head in comparative imaging studies. There is therefore an unmet need for evidence synthesis to guide evidence-based decisions in the selection of radiotracers. The objective of this study was therefore to assess the detection rate of various radiotracers for the rPC.

**Methods:**

The PUBMED, EMBASE, and the EU and NIH trials databases were searched without date or language restriction for comparative imaging tracers for 13 radiotracers of principal interest. Key search terms included 18F-PSMA-1007, 18F-DCPFyl, 68Ga-PSMA-11, 18F-PSMA-11, 68Ga-PSMA-I&T, 68Ga-THP-PSMA, 64Cu-PSMA-617, 18F-JK-PSMA-7, 18F-Fluciclovine, 18F-FABC, 18F-Choline, 11C-Choline, and 68Ga-RM2. Studies reporting comparative imaging data in humans in rPC were selected. Single armed studies and matched pair analyses were excluded. Twelve studies with eight radiotracers were eligible for inclusion. Two independent reviewers screened all studies (using the PRISMA-NMA statement) for inclusion criteria, extracted data, and assessed risk of bias (using the QUADAS-2 tool). A network meta-analysis was performed using Markov-Chain Monte Carlo Bayesian analysis to obtain estimated detection rate odds ratios for each tracer combination.

**Results:**

A majority of studies were judged to be at risk of publication bias. With the exception of 18F-PSMA-1007, little difference in terms of detection rate was revealed between the three most commonly used PSMA-radiotracers (^68^Ga-PSMA-11, ^18^F-PSMA-1007, ^18^F-DCFPyl), which in turn showed clear superiority to choline and fluciclovine using the derived network.

**Conclusion:**

Differences in patient-level detection rates were observed between PSMA- and choline-radiotracers. However, there is currently insufficient evidence to favour one of the four routinely used PSMA-radioligands (PSMA-11, PSMA-1007, PSMA-I&T, and DCFPyl) over another owing to the limited evidence base and risk of publication bias revealed by our systematic review. A further limitation was lack of reporting on diagnostic accuracy, which might favour radiotracers with low specificity in an analysis restricted only to detection rate. The NMA derived can be used to inform the design of future clinical trials and highlight areas where current evidence is weak.

**Supplementary Information:**

The online version contains supplementary material available at 10.1007/s00259-021-05210-9.

## Introduction

PSMA-radiotracers are increasingly utilized for the investigation of biochemical recurrence of prostate cancer (rPC) replacing previous generation radiotracers [1]. Given the increasing importance of nuclear medicine imaging for radiation treatment planning [2], the challenge is to identify the optimum radiotracer for the accurate detection of disease. Despite the fact that PSMA-radiotracers are now well established, agreement for their reimbursement, approval for their use or their inclusion in guidelines has hitherto been limited [3]. Despite the preponderance of data supporting their use, the European Association of Urology (EAU) guidelines rate their recommendation for PSMA-PET/CT in the setting of biochemical recurrence post radical prostatectomy as “weak”, and where PSMA-PET/CT is not available, fluciclovine-PET/CT is endorsed. There is a paucity of “gold standard” comparative imaging trials available to verify claims made for the utility of PSMA-PET/CT [4]. Consequently, the issue of which of the multitude of radiotracers is optimal remains the subject of debate [5, 6], and choline tracers remain standard of care in many locations [7]. Even in locations where PSMA-PET/CT is available, disparities in access are encountered [8, 9], implying that more robust evidence is needed to improve access to PSMA-PET/CT for men with rPC.

High-quality synthesis of the available evidence is therefore an important unmet need in the imaging of rPC. While a number of meta-analyses are published for imaging of rPC [10–14], few studies address underlying deficiencies in the data where studies are few in number, small in size, of designs which are at risk of bias and where few radiotracers have been tested in head-to-head comparative studies. Network meta-analysis (NMA) is a useful technique which affords indirect data and may provide greater statistical precision than pairwise meta-analysis [15]. The aim of this NMA is to provide a systematic evidence synthesis in the nuclear imaging of rPC and seeks to evaluate the performance of available radiotracers.

## Materials and methods

The study design and methodology were established using EQUATOR guidelines, using the preferred reporting items for systematic review and meta-analysis statement as adapted for network meta-analyses (PRISMA-NMA) [16]. This study was registered with PROSPERO (Centre for Reviews and Dissemination, identification number ID203299) where no comparable network meta-analyses were previously registered.

### Search strategy and data extraction

The PUBMED and EMBASE databases were searched for studies reporting direct comparisons of PC-specific radiotracers. Supplementary searches were performed using the EU and NIH trials databases. Thirteen PET-radiotracers of interest were chosen for inclusion: ^18^F-PSMA-1007, ^18^F-DCPFyl, ^68^Ga-PSMA-11, ^18^F-PSMA-11, ^68^Ga-PSMA-I&T, ^68^Ga-THP-PSMA, ^64^Cu-PSMA-617, ^18^F-JK-PSMA-7, ^18^F-Fluciclovine, ^18^F-FACBC, ^18^F-Choline, ^11^C-Choline, and ^68^Ga-RM2. Single photon emission computed tomography (SPECT) tracers and bone-specific tracers (e.g. ^18^F-NaF) were not considered in this analysis. A contingency table for all possible combinations of search terms was made, and using Boolean logic, the databases were interrogated using a sensitive search string for studies which included all combinations of two of these tracers as keywords or in their titles. All relevant synonyms were included (e.g. PSMA-11, also known as PSMA-HBEDD, PSMA-DFKZ; ^18^F-Choline, also known as fluorocholine, FECH, ^18^F-Fluciclovine, also known as ^18^F-FACBC) and the string allowed for variation in radiolabelling (e.g. ^64^Cu-PSMA-617, ^68^Ga-PSMA-617). The full string is provided in supplementary materials. No date or language restriction was imposed. The search and article selection were performed by two independent evaluators (IA and CM). Disagreements were resolved by consensus in discussion with a third evaluator (AAO) and recorded in the study database.

### Study selection

Both evaluators screened the titles and abstracts of all search results. No language or date restrictions were used. Inclusion criteria were studies reporting direct head-to-head comparisons of radiotracers in rPC in human beings. Only comparative (intra-individual) imaging studies were included, and single-arm trials or matched pair analyses were excluded. Randomized control trials, cohort studies, and case control and cross-sectional studies were included. Studies which reported data for primary prostate cancer only, PET/MRI studies restricted only to the pelvis, matched pair data, case reports or series, those with small sample sizes (<10 patients), bioavailability or pharmacodynamic studies, and studies not performed in humans were excluded from the analysis.

### Outcome measures and data extraction

The primary outcome was the patient-level detection rate of the radiotracer, defined as the proportion of patients tested who have a pathological or “positive” PET/CT (“detection rate”). Secondary outcomes were lesion-based sensitivity, specificity, positive and negative predictive values for the detection of rPC, and semi-quantitative parameters (standardized uptake values SUV and lesion to background contrast ratio for pathological lesions). Data were recorded by two reviewers (IA recording the data, CM second double-checking).

The following patient-level and study effect modifiers were recorded to facilitate possible meta-regression for covariates: prostate-specific antigen value (PSA), age, tumour stage (TNM), and Gleason score (GS). Ongoing androgen deprivation therapy (ADT), which is known to make PSMA-radiotracer uptake unpredictable [17], uptake time, injected radiotracer activity, time interval between the two scans, and prior treatment (radiotherapy and/or prostatectomy) were recorded.

### Data synthesis and statistical analysis

A Bayesian meta-analysis using a single summary statistic (the relative detection rate odds ratio as primary outcome) was performed using open source, peer-reviewed software (NetMetaXL, Canadian Agency for Drugs and Technologies in Health [18]). As heterogeneity between different trials due to the deviating protocols and patients characteristics can be expected, a random effects model was used with a Markov-Chain Monte Carlo Bayesian analysis software WinBUGS (Vers 1.4.3, Medical Research Council Biostatics Unit [19]), which we accessed via NetMetaXL. Following recommendations for NMA in sparsely informed networks [18, 20, 21], we relied on the informative priors derived by Turner et al. [22] for the variance of the between-trial heterogeneity, while we used vague priors for all other parameters (N(0, 10,000)) as previously published [23]. We run the estimation with a burn-in of 10,000 and sampling 10,000 iterations from three chains. Convergence of iterations was assessed using the Gelman-Rubin-Brooks statistic (Supplementary Materials). Graphs were drawn in Excel (Vers. 2016, Microsoft). Studies were ranked according to SUCRA values (surface under the cumulative ranking curve). The SUCRA value is a numerical representation of an intervention’s overall ranking and is expressed as a percentage. The higher the score, the higher the probability that an intervention is ranked higher compared to another [24].

### Risk of bias, quality of evidence, and analysis of network heterogeneity

Risk of bias was assessed using the revised Quality Assessment of Diagnostic Accuracy Studies Tool (QUADAS-2) by two individual evaluators with discrepancies resolved by consensus (IA and CM).

Model convergence is assessed in NetMetaXL using the Brooks-Gelman-Rubin method and by checking whether the Monte Carlo error is less than 5% of the standard deviation of the effect estimates and between-study variance as previously described [18]. Network transitivity was assumed through the presumption of no systematic differences in the assignment of radiotracers within comparative imaging trials and their theoretical joint randomizability. Comparison between direct and indirect evidence was by means of closed loop analysis.

## Results

### Study selection

**Fig. 1:**
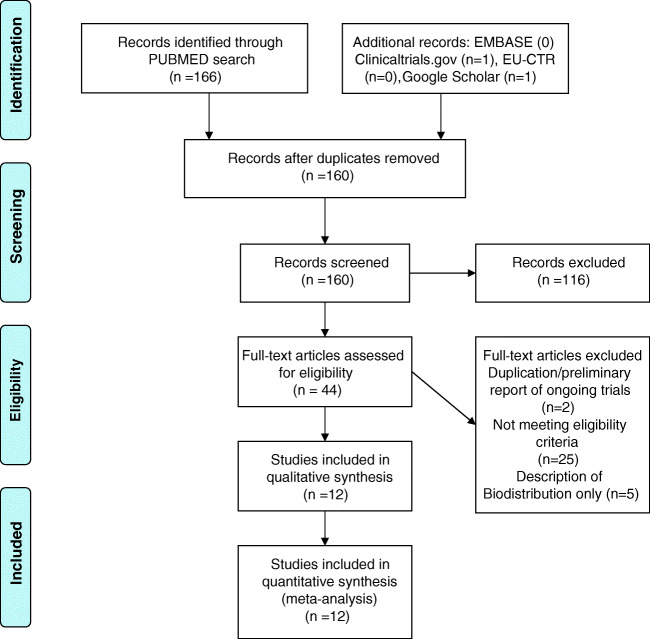
PRISMA flowchart for literature search and selection

We screened 166 titles and abstracts and 44 full text article finding twelve studies with 1356 patients and eight radiotracers meeting our inclusion criteria. Study characteristics are outlined in Table 1, and the PRISMA flow diagram is shown in Fig. 1.

**Table 1:** Study details and cohort characteristics

Study author	Tracer 1	Tracer 2	PSA	GS	ADT	Treatment	Time gap (days)	Patients	Activity 1	Activity 2	Time scan 1	Time scan 2
Cantiello 2018 [25]	^64^Cu-PSMA-617	^18^F-FCH	0.8*	NR	NR	OP, no RT	15	43	315	314	60	60
Dietlein 2015 [26]	^68^Ga-PSMA-11 (HBED)	^18^F-DCFPyl	6.33	NR	NR	RT/OP	21	14	128	318	120	120
Pernthaler 2019 [27]	^68^Ga-PSMA-11 (HBED)	^18^F-Fluciclovine	14.9	7.7	Yes	RT/OP	22	58	170	370	60	3
Witkowska 2019 [28]	18F-PSMA-1007	^18^F-FCH	0.77	7.1	Yes	RT/OP	54	40	296	248	95	87
Morigi 2015 [29]	^68^Ga-PSMA-11 (HBED)	^18^F-FCH	1.72	7	Yes	RT/OP	30	38	2/Kg	3.5/Kg	45	20
Afshar-Oromieh 2014 [30]	^68^Ga-PSMA-11 (HBED)	^18^F-FCH	11.1	7.4	Yes	RT/OP	30	37	2/Kg	241	60	60
Calais 2019 [4]	^68^Ga-PSMA-11 (HBED)	^18^F-Fluciclovine	0.48	NR	Yes	OP, no RT	15	50	139	381	61	61
Schwenck 2017 [31]	^68^Ga-PSMA-11 (HBED)	^11^C-Choline	9.47	7.7	NR	RT/OP	1	103	166	625	60	5
Emmett 2019 [32]	^68^Ga-PSMA-11 (HBED)	^18^F-FCH	0.42*	7.3	Yes	RT/OP	14	31 / 91	2/Kg	3.6/Kg	60	60
Nanni 2015 [33]	^18^F-Fluciclovine	^11^C-Choline	3.2	7.2	Yes	RT/OP	7	50	340	3.4/Kg	3.6	3.5
Nanni 2016 [34]	^18^F-Fluciclovine	^11^C-Choline	6.99	NR	Yes	RT/OP	7	89	370	3.4/kg	4	4
Bluemel 2017 [35]	^68^Ga-PSMA-I&T	^18^F-FCH	5.4	NR	NR	RT/OP	NR	125	311	133	60	60

NR means either not reported, or because (partially) missing data could not be calculated. PSA reported as mean, data with asterisk as median (mean not reported), *GS* Gleason score, ADT (yes/no patients with androgen deprivation therapy included), Cohort (RT, post-radiotherapy; OP, post-prostatectomy), time gap (time between examination 1 and 2 in days), patients (number of patients included in both arms), activity (mean activity of radiotracer 1 and 2 applied in MBq or, where not recorded, reported target dose MBq/kg), time scan 1 and scan 2 p.i. (time of scan acquisition post injection of radiotracer in minutes)

### Reporting of outcome measures

All studies reported rate of pathological scans (patient-level “detection rate”) per trial arm, or this could be inferred from the reported data. Statistically pre-defined endpoints were reported only for one study [4]. Quality of evidence reporting was largely poor in the included studies. For studies of retrospective design, no adequate explanation was given for why individual patients had undergone imaging with two tracers; few reported details of patient exclusion. The majority of studies of retrospective design do not describe how results of the index test are interpreted separate to the reference test, pre-defined reference standards, or whether all patients were included in the analysis. Semi-quantitative standardized uptake values (SUV) were reported by only seven studies [4, 26, 28, 30, 31, 33, 34], and definition of uptake (SUVmax/SUVmean, thresholds and definition) was heterogeneously reported. Only one study reports diagnostic accuracy data [4], and two studies used a defined reference standard for lesion confirmation or interpretation [4, 32]. Likewise, reporting of secondary effect modifiers was incomplete: e.g. with two studies reporting only the median PSA [25, 32], whereas the remainder report the mean. Only one study stratified patients by previous treatment [36]. The limited and incomplete reporting of patient covariates means that no statistical model could be fitted or meta-regression performed, and only a descriptive analysis (Table 1) could be performed.

### Network structure and results of individual studies

**Fig. 2:**
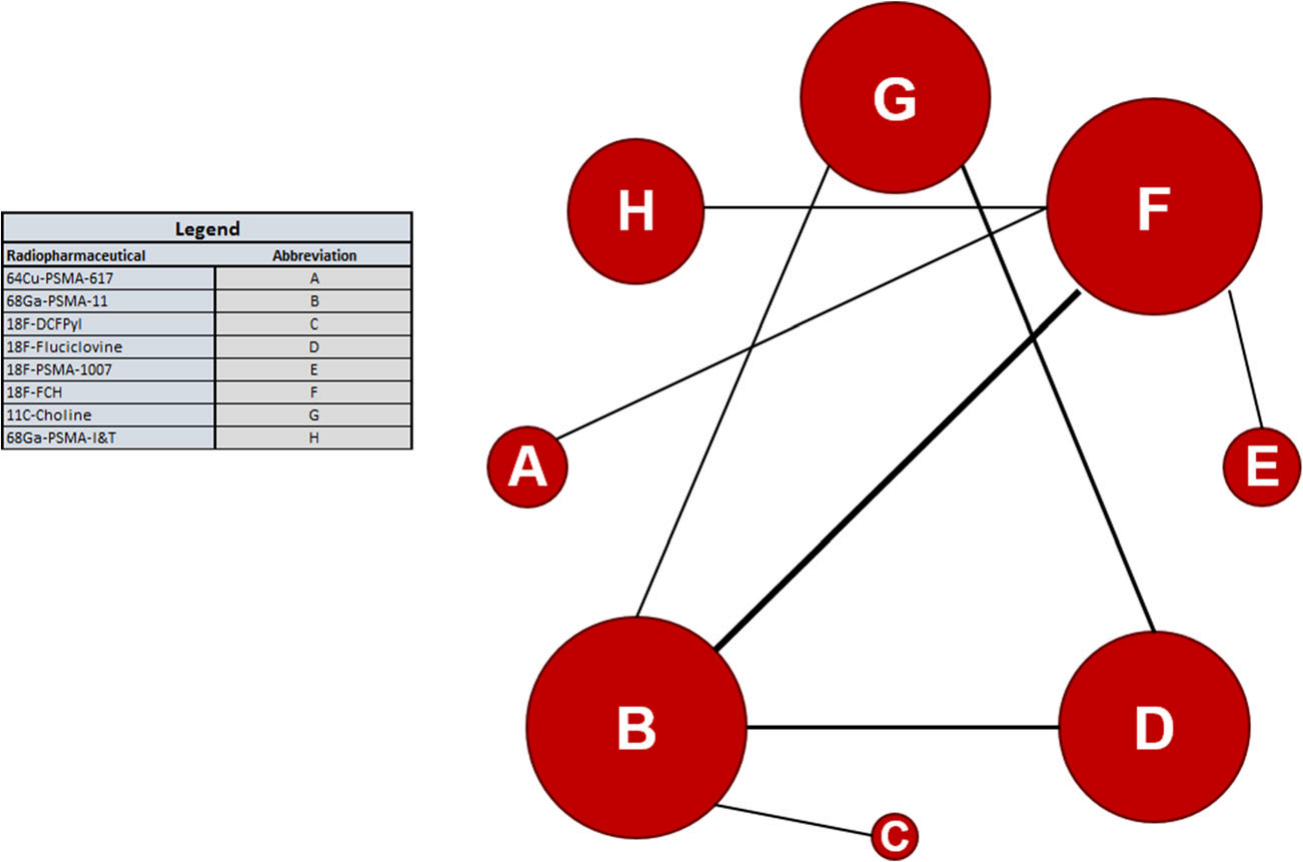
The network created by the included studies. The area of the node represents the number of patients in each trial; the thickness of the edge represents the number of studies. The distances are only representative

The network given by the analyses is shown in Fig. 2. Whereas all studies reported the primary outcome (rate of pathologic PET-scans), reporting of secondary outcomes was limited, and reporting of effect modifiers was also incomplete (four studies did not report ADT status [25, 26, 31, 35]; four did not report Gleason score [25, 26, 33, 35, 36]; see Table 1).

### Network meta-analysis results

**Fig. 3:**
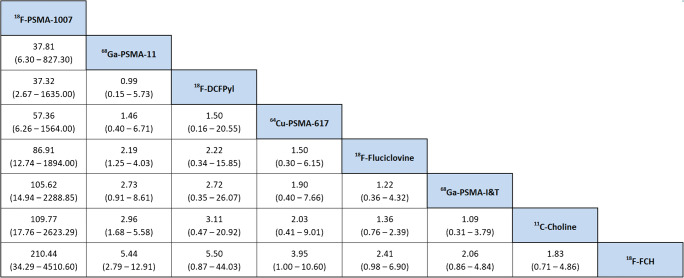
Detection rate ratios (odds of pathological PET in tracer A compared to B) for pairwise comparisons, ordered by SUCRA (random effects, informative). Ratios > 1 imply that the radiotracer of comparison which is left most has a greater detection rate

**Fig. 4:**
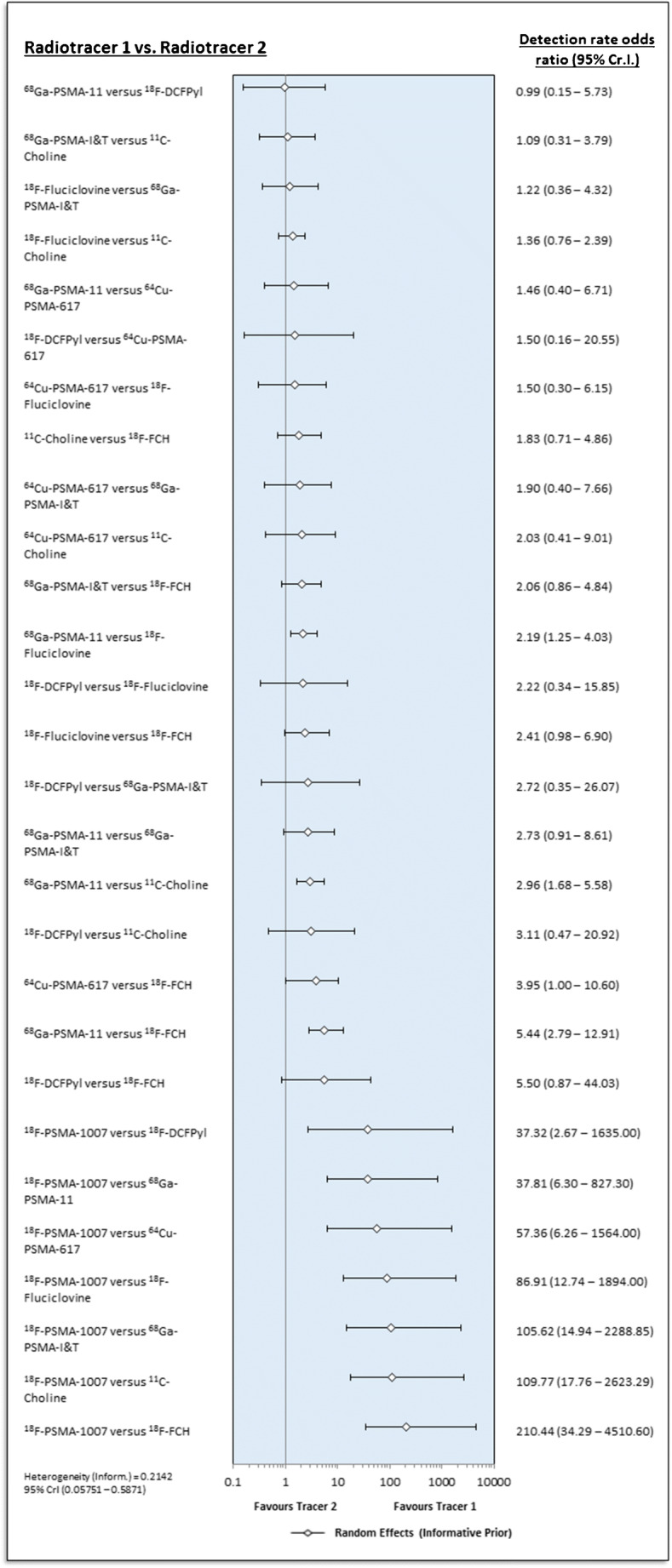
Forest plot comparing different radiotracers, including inferred comparisons from the network (random effects with informative priors)

The estimated ratios of the rate of pathologic PET-scans for each pairwise comparison between radiotracers of the NMA are presented in Fig. 3, ordered by the SUCRA values. The respective SUCRA values are reported in Table 2. In Fig. 4, we present forest plots for all radiotracer comparisons. Broadly, when considering the SUCRA values (reported in supplementary Table S2), the three most commonly used PSMA-radiotracers (PSMA-11, PSMA-1007 and DCFPyl) are the three radiotracers most likely to be ranked first with respect to estimated detection rate. On analysis of the forest plots, there appears to be little difference in the pairwise comparison of ^68^Ga-PSMA-11 and ^18^F-DCFPyl. ^18^F-PSMA-1007 is favoured in all pairwise comparisons, albeit with very wide credible confidence limits, reflecting underlying uncertainty in the derived network owing to the small number of studies available (see Fig. 4). For many comparisons (27 out of 36 total pairwise comparisons), the credible intervals for the estimated rate of pathologic PET-scans lie athwart 1.0, meaning that neither tracer for these comparisons can be formally favoured.

**Table 2:** Surface under the cumulative ranking curve (SUCRA) for the various radiotracers

Radiotracer	SUCRA
^18^F-PSMA-1007	0.9997
^68^Ga-PSMA-11	0.7385
^18^F-DCFPyl	0.6607
^64^Cu-PSMA-617	0.5626
^18^F-Fluciclovine	0.4242
^68^Ga-PSMA-I&T	0.3303
^11^C-Choline	0.2518
^18^F-FCH	0.03219

### Assessment of quality of evidence

**Fig. 5:**
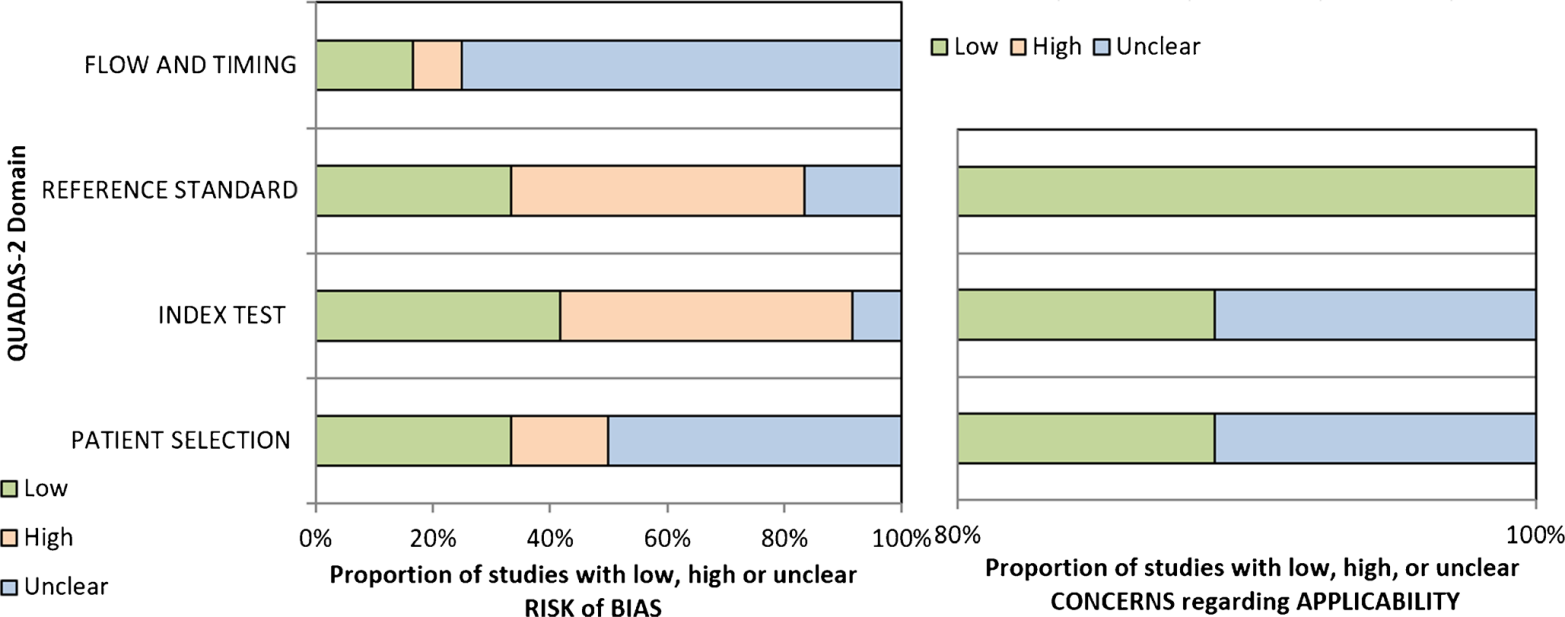
Proportion of studies at low, high, or unclear risk of bias and regarding applicability

QUADAS-2 revealed no study to be at high overall risk of bias. Ten were judged as at being risk of bias, and the remaining two studies were judged to be at overall low risk of bias [4, 32]. Only one study conformed to acceptable guidelines for reporting trials (CONSORT) [4]. The results are shown in Fig. 5. Only one study was judged to be at risk of non-applicability [35], with the remainder at low risk of non-applicability. The individual results per study are in supplementary materials.

### Assessment of model reliability and heterogeneity

**Fig. 6:**
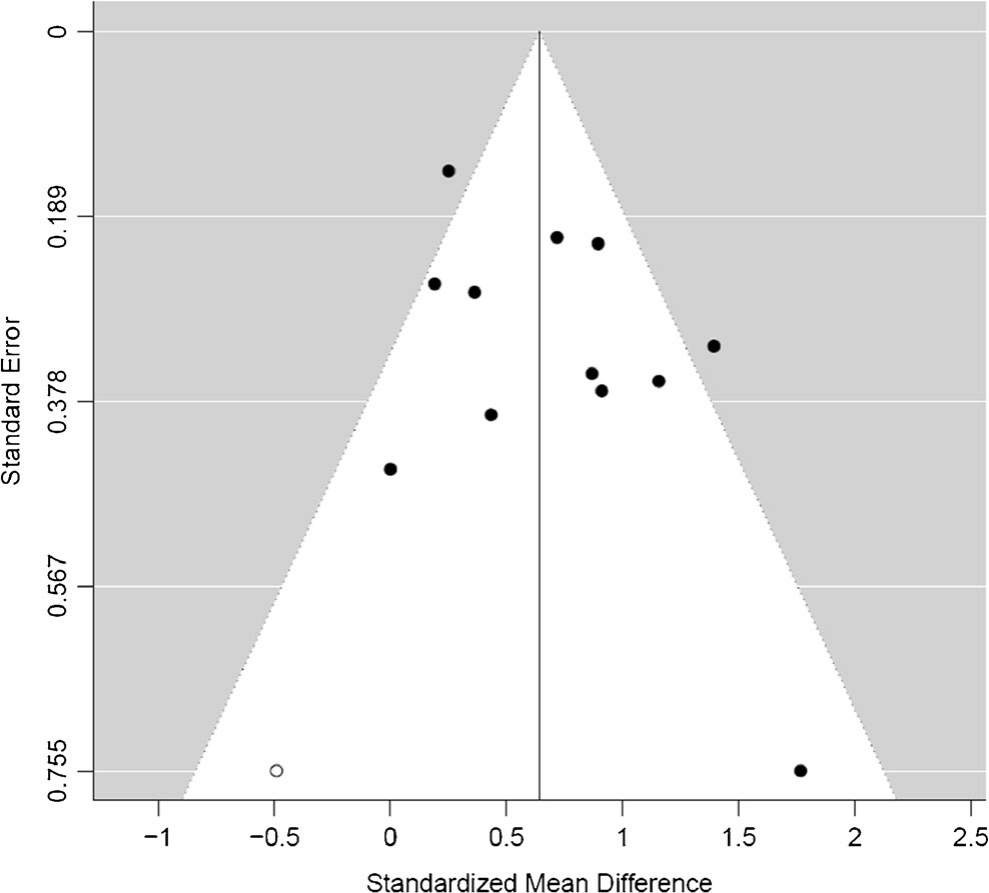
Funnel plot showing PET positivity rate (observed outcome) versus the standard error in the detection rate (*p* = 0.05) where the x-axis represents standard error and the y-axis detection rate, and each dot represents each study. A number of studies fall outside the 95% control limits and are at risk of small study effects

In Fig. 6, we show a Begg’s funnel plot, with derived standard error in the detection rate versus the detection rate reported for the informative fixed model. Study heterogeneity was assessed as moderate (*I*^2^=58.21%, *p*=0.0072). Overall, study symmetry was observed with no funnel plot asymmetry (Kendal’s Tau = 0.2121, *p*=0.3807). The fail-safe N was high (*N*=267) suggesting that the meta-analysis was not susceptible to publication bias.

**Fig. 7:**
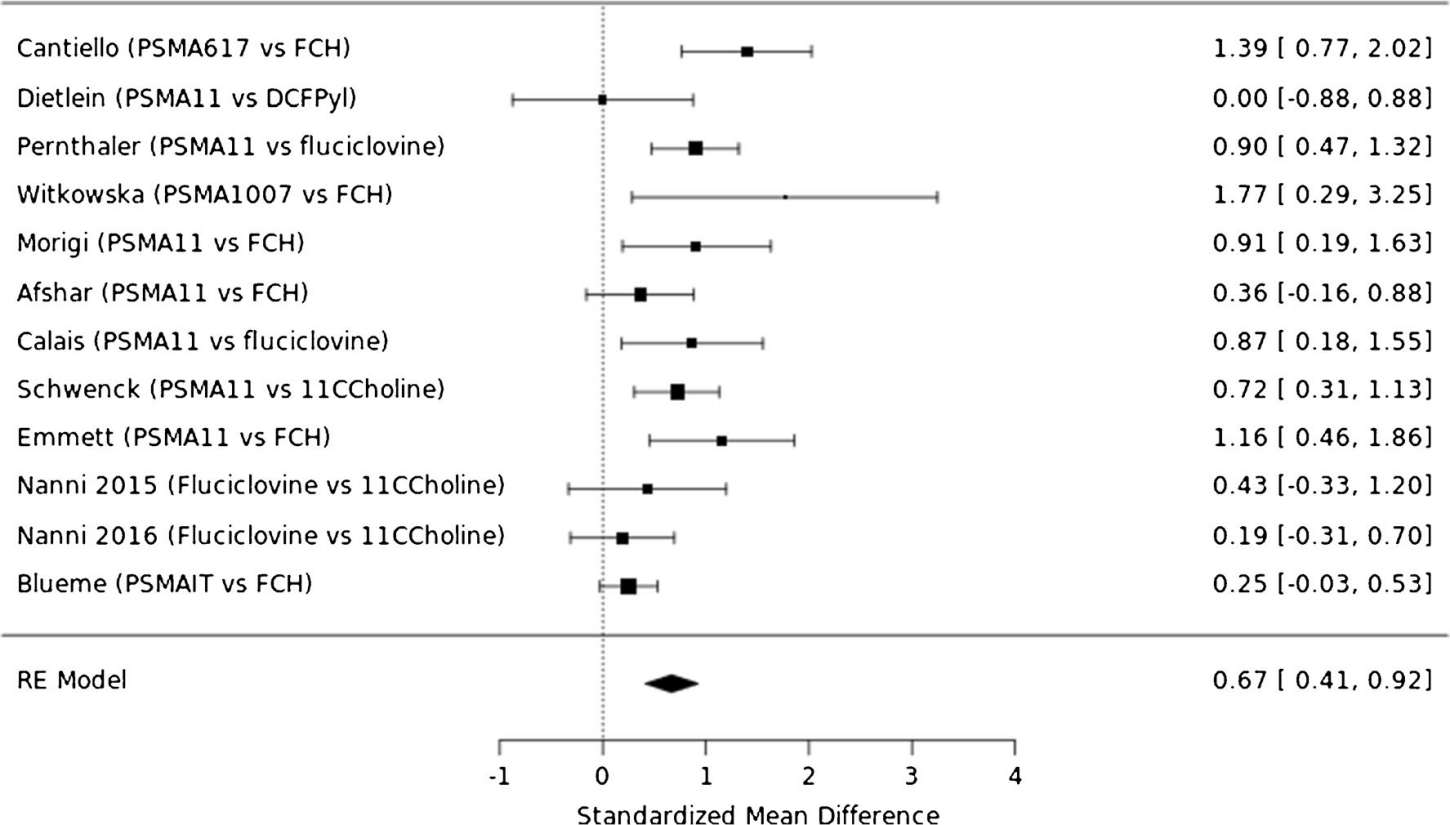
Forest plot for fixed effects model, pairwise comparison of direct data

In the identified network, one closed loop exists by following the path ^68^Ga-PSMA-11 over ^11^C-Choline and ^18^F-Fluciclovine back to ^68^Ga-PSMA-11. We used a node-split model to compare results from the NMA with and without the direct evidence for each of the edges and found no evidence on statistical inconsistency in any of the investigated contrasts suggesting agreement between the direct and indirect evidence of the NMA (Supplementary Fig. S2). To aid comparison of direct and indirect data, a forest plot for direct pairwise comparisons is shown in Fig. 7.

## Discussion

This NMA is, to our knowledge, the first to consider all available comparative imaging data for PSMA and non-PSMA-based radiotracers in recurrent prostate cancers using a networked meta-analysis approach, confirmed by a search of the PROSPERO data-base, thus representing the most comprehensive review of available comparative radiotracer imaging trials for rPC. Although a large amount of robust data has been collected supporting the use of PSMA-radiotracers, including (non-network) meta-analyses, for example, for ^68^Ga-PSMA-11 [12, 13, 37] and, more recently, for ^18^F-based radiotracers [38, 39], only few prospective comparative imaging trials have been performed to confirm this. As a result, the role for PSMA-radiotracers remains a topic of controversy [5, 6], and a significant evidence “gap” has emerged between the accumulated experience in nearly a decade of routine PSMA-radiotracer use and the pronouncements of guidelines and regulatory authorities. Consequently, implementation or reimbursement of PSMA-PET/CT for rPC is not ubiquitous and in many centres where choline-based radiotracers remain standard of care. Furthermore, when choosing between the plethora of radiotracers available, the referring oncologist, urologist, or the nuclear medicine physician have little objective evidence upon which to favour one radiotracer over another.

It is in this clinical context that we interpret our data which show clear superiority of the three most established PSMA-based radiotracers (^68^Ga-PSMA-11, ^18^F-PSMA-1007 and ^18^F-DCFPyl) when compared to previous generation choline-based tracers. These findings further strengthen the argument for replacement of choline with PSMA-radiotracers. Additional weight is lent to these findings by previously published systematic analyses, for example comparing PSMA-radiotracers to fluciclovine [10]. Our analysis also confirms the high detection rate of both ^18^F-labelled PSMA-radiotracers shown in the previous (non-networked) meta-analyses (0.71–0.88 by Treglia et al. [14]) and ^68^Ga-PSMA-11 (0.74 ± 95% confidence interval 0.55–0.70 for PSA >2.0 and 0.94 ± 95% confidence interval 0.91–0.96 PSA >2.0 Hope et al. [11]). The strength of using an NMA approach is the ability to pool all available data and combine direct and indirect evidence. Whereas published data (direct evidence) only affords 12 pairwise comparisons between radiotracers, a NMA incorporating indirect evidence affords a comparison between all radiotracers (36 pairwise comparisons in total) and is a known strength of NMA [40].

The NMA reveals an inferior estimated detection rate for ^68^Ga-PSMA-I&T compared to ^18^F-Fluciclovine, whereas ^64^Cu-PSMA-617 is superior to the latter. This result finds partial explanation in the pharmacokinetics of these two theragnostic radiotracers, which have different dynamics than the diagnostic tracers PSMA-11, PSMA-1007, and DCFPyL. The longer half-life of ^64^Cu (12.7 h [41]) may facilitate improved lesion contrast. Further studies with different radiolabelled tracers are required to explore the true potential of these various ligands.

The NMA reveals comparable performances for ^18^F-DCFPyl and ^68^Ga-PSMA-11. When considering both SUCRA rankings (where ^18^F-PSMA-1007 has the highest probability to be ranked highest) and the estimated pairwise detection rate odds ratios, ^18^F-PSMA-1007 is favoured by our NMA. However, we interpret this finding with due caution [24], particularly in light of the fact that only one comparative imaging study was available for ^18^F-PSMA-1007 [28], which was underpowered with only 40 patients and was judged to be at risk of publication bias by the QUADAS-2 tool (c.f. Table S1 in supplementary materials). In principle, multiple studies are required to demonstrate reproducibility, and these can then be synthesized by meta-analysis. The strength of the NMA is that, through the incorporation of direct and indirect evidence, overall certainty in this single trial result can be expressed quantitatively and in a wider context through a survey of all comparative imaging data available. As such, the wide credible confidence intervals, for example, for the pairwise comparison of ^18^F-PSMA-1007, ^18^F-DCFPyl, or ^68^Ga-PSMA-11, reflect the overall lack of certainty in this result.

Furthermore, we also urge nuanced interpretation of this finding in the light of other available evidence: Dietlein et al. report data for 27 individuals who, following initial equivocal or negative ^68^Ga-PSMA-11, ^18^F-DCFPyL, or ^18^F-JK-PSMA-7 PET/CT, underwent additional ^18^F-PMSA-1007 (this study did not meet the inclusion criteria for this NMA owing to inability to separate the comparators). A retrospective matched pair analysis by Rauscher et al. compared ^18^F-PSMA-1007 and ^68^Ga-PSMA-11 [26]. Importantly, neither of these studies reported any increased detection rate for ^18^F-PSMA-1007. Although a high detection rate for ^18^F-PSMA-1007 is reported by Rahbar et al. [38], less favourable results are found when comparing the prospectively obtained data of Witkowska et al. [42] for ^18^F-PSMA-1007 with ^68^Ga-PSMA-11 (e.g. for PSA <0.5, the detection rates were 35% and 38%, respectively, favouring ^68^Ga-PSMA-11). Likewise a comparison of the retrospective data of Giesel et al- [39] for ^18^F-PSMA-1007 with those of Fendler et al. [43] for ^68^Ga-PSMA-11 reveals a detection rate favouring the latter (81.3 vs 84%). Therefore, our NMA reveals comparative imaging trials with ^18^F-PSMA-1007 to be an area of unmet need are urgently required before any evidence-based recommendations can be made for this radiotracer.

The estimated superiority of ^11^C-Choline compared to ^18^F-FCH is a surprising result. This result could only be revealed by NMA, since no comparative imaging studies were identified by our systematic literature search for any pair of choline-based tracers. ^11^C has a higher positron energy compared to ^18^F (390 vs. 252 MeV) with a higher positron range (1.27 vs. 0.66 mm [41]) which is theoretically at detriment to the image quality. The wide credible confidence intervals in the forest plot suggest that further studies are required to resolve which of these two radiotracers indeed show the greatest detection rate.

In addition to the caveats stated, we urge an appreciation of the underlying methodology and its limitations to avoid the drawing of erroneous conclusions, particularly given the large number of studies which are at risk of bias [24]. Moreover, detection rate or patient-level sensitivity is not, and should not be, the sole criterion for radiotracer choice. Significantly, Rauscher et al. revealed fivefold greater numbers of uncertain lesions for ^18^F-PSMA-1007 compared to ^68^Ga-PSMA-11 [44], corroborated by Witkowska’s finding of a lower positive predictive value (67%) for^18^F-PSMA-1007 [42] than that found by Fendler et al. for ^68^Ga-PSMA-11 (84% verified by histology [43]) or the meta-analysis of Perera et al. for ^68^Ga-PSMA-11 (specificity 99% [13]). This represents an important drawback for ^18^F-PSMA-1007 and which cannot be taken into account by any NMA until such a time as further comparative diagnostic accuracy studies are published.

Finally, there are a myriad of considerations which are of equal importance when considering a radiotracer beyond the estimated detection rate revealed by our NMA. For example, given the high cost and complexity of infrastructure required to instate a PSMA-imaging program, cost efficacy is an important consideration. The cost efficacy for ^18^F radiotracers has been posited by some authors as a reason justifying their use [39], although we find no such systematic study of this issue which would corroborate this assertion. Assessment of diagnostic accuracy is mandatory when considering any diagnostic test. We find only one comparative imaging study reporting diagnostic accuracy to follow-up using a defined composite reference standard. Whereas single-arm diagnostic accuracy studies with a reference standard and statistically defined endpoints are available for the ^68^Ga-PSMA-11 [43] and ^18^F-DCFPyl [45], this is not the case for other tracers.

Although our quantitative assessment of study bias, heterogeneity, and the fail-safe *N* calculation revealed low vulnerability of the NMA to publication bias and analysis of closed loops within the network revealed concordance between direct and indirect evidence leads to overall confidence in our network, we nevertheless note that the number of studies judged to be at risk of bias by the QUADAS-2 tool was high. The studies included were largely underpowered and thus at risk of small study effects, a phenomenon where smaller studies show larger treatment effects than large ones or being underpowered to test hypotheses with any precision [23]. We recognize that the small patient numbers is a result of the difficulties in performing comparative imaging studies with radiotracers.

As a result, a number of pairwise comparisons are precluded by wide credible confidence intervals. Although this can be viewed as a study limitation, we recall that this paucity of information was indeed the motivation for this study: NMA can aid real-world decision-making where data is limited and imperfect by facilitating a consideration of underlying heterogeneity in the data [22] and incorporation of indirect data [40] and can afford improved design of future studies by highlighting areas where studies of greater precision are required or which pairs of tracers should be studied in order to improve overall conclusions based on systematic evidence syntheses [46].

Transitivity is a pre-requisite for any NMA; i.e. all study populations are comparable. We therefore exclude comparative imaging studies for primary staging of PC. Ideally, studies would be stratified on the basis of prior therapy. As shown in Table 1, this was done for very few studies. PSA values were highly heterogeneous ranging from a mean of 0.42 to 14.9. We therefore use a random effects model to compensate for heterogeneity in patient-level effect modifiers. A number of parameters can contribute to the likelihood of a positive PET/CT in rPC, and these would ideally be controlled and directly comparable between cohorts: the prostate-specific antigen level (PSA) and clinical setting (post-radiotherapy or post-prostatectomy) were all identified as independent predictors of a positive PSMA-PET/CT [47]. Androgen deprivation therapy (ADT) is known to modulate PSMA-expression and can make the results of a PSMA-PET/CT unpredictable [17]. Furthermore, the relationship between the patient-level detection rate (rate of pathological or “positive” PET scans) and PSA is non-linear [37], meaning that the expected effect size between two tracers can differ: a study restricting itself to early biochemical recurrence can therefore report a greater apparent effect size between PSMA-radiotracers and choline-radiotracers, for example, than one which includes patients at later stages of recurrence. As shown in Table 1, few studies adequately report these parameters, making a planned meta-regression for these covariates non-feasible. Examinations would be performed under the same and, ideally, optimized clinical conditions. For example, later acquisitions show improved lesion detection for some PSMA tracers [48, 49]; the time-point of image acquisition should therefore be standardized. None of the studies report the use of a diuretic for tracers undergoing renal excretion such as ^68^Ga-PSMA-11 or ^18^F-DCFPyL, a simple and low-cost clinical manoeuvre which can increase lesion detection [50, 51] and is an important consideration in designing a fair comparison of renal with non-renal excreted tracers. Finally, we wish to highlight the point that no PSMA-radiotracer has yet demonstrated a 100% sensitivity, and non PSMA-avid rPC is a potentially underreported phenomenon. No trial data is available for optimal imaging sequence in the scenario of PSMA-negative rPC, where additional PET/CT with choline can be of diagnostic benefit [52]. Finally, any future studies aspiring to inform evidence-based decision-making for PSMA-radiotracers should be designed in the context of existing evidence; this NMA provides valuable information about where studies with larger statistical precision are required and highlights which radiotracers should be compared in order to improve any future systematic network analysis [46] and where more multicentre studies might be a strategy to increase the statistical power of future comparative imaging studies with radiotracers.

## Conclusion

Our NMA compares all comparative imaging data for PET-radiotracers in rPC. Our findings confirm the superiority of the three most commonly used PSMA-radiotracers (^68^Ga-PSMA-11, ^18^F-PSMA-1007, and ^18^F-DCFPyl), particularly when compared to previous generation choline-based tracers. By using a networked approach, we are able to provide an analysis of the available literature in its totality, rather than restricting our analysis to single radiotracers, a method that may be of greater reliability. This method also allows comparisons between radiotracers where no direct head-to-head comparisons exist. We find insufficient evidence to favour one particular PSMA-radiotracer over another and large overlap between ^68^Ga and ^18^F-labelled PSMA-radiotracers with regard to patient-level detection rates. Our study also highlights some deficiencies in standards of reporting of studies involving radiotracers and the necessity for their standardization.

## Supplementary information

ESM 1 (DOCX 96 kb)

## Declarations

The study was performed in accordance with the Helsinki declaration. No approval from an ethics committee was required.
