# Correction to: Comparing the diagnostic performance of radiotracers in recurrent prostate cancer: a systematic review and network meta-analysis

**DOI:** 10.1007/s00259-021-05300-8

**Published:** 2021-03-22

**Authors:** Ian Leigh Alberts, Svenja Elizabeth Seide, Clemens Mingels, Karl Peter Bohn, Kuangyu Shi, Helle D. Zacho, Axel Rominger, Ali Afshar-Oromieh

**Affiliations:** 1 Department of Nuclear Medicine. Inselspital, Bern University Hospital, University of Bern, Street: Freiburgstr. 18, CH-3010 Bern, Switzerland; 2 Institute of Medical Biometry and Informatics, University of Heidelberg, Im Neuenheimer Feld 130.3, 69120 Heidelberg, Germany; 3 Department of Nuclear Medicine and Clinical Cancer Research Center, Aalborg University Hospital, Hobrovej 18-22, DK-9000 Aalborg, Denmark

**Correction to: Eur J Nucl Med Mol Imaging**

10.1007/s00259-021-05210-9

An error at the production stage included unnecessary software dialogue boxes in figs. 2 and 4 which has been corrected. The content of the images otherwise remains the same.

**Fig. 2:**
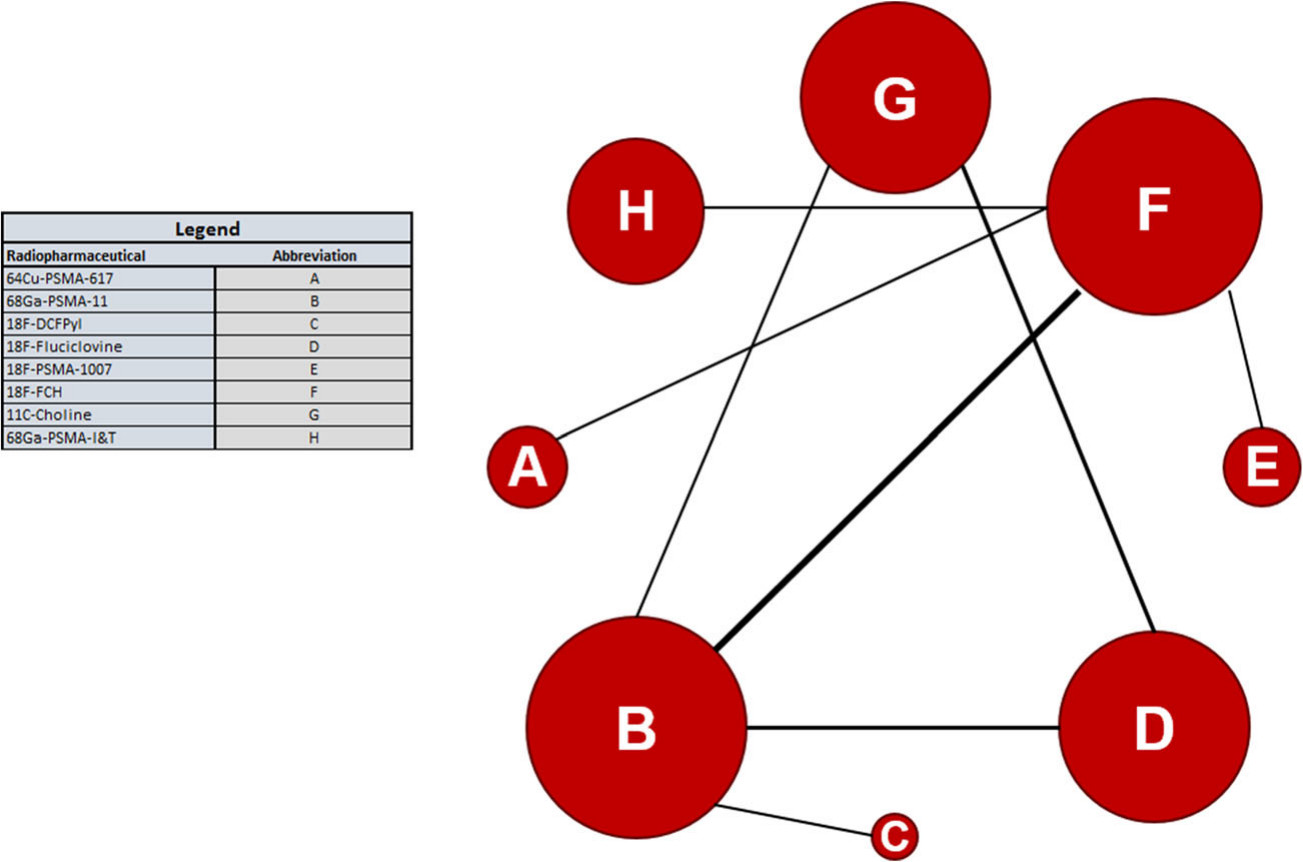
The network created by the included studies. The area of the node represents the number of patients in each trial; the thickness of the edge represents the number of studies. The distances are only representative

**Fig. 4:**
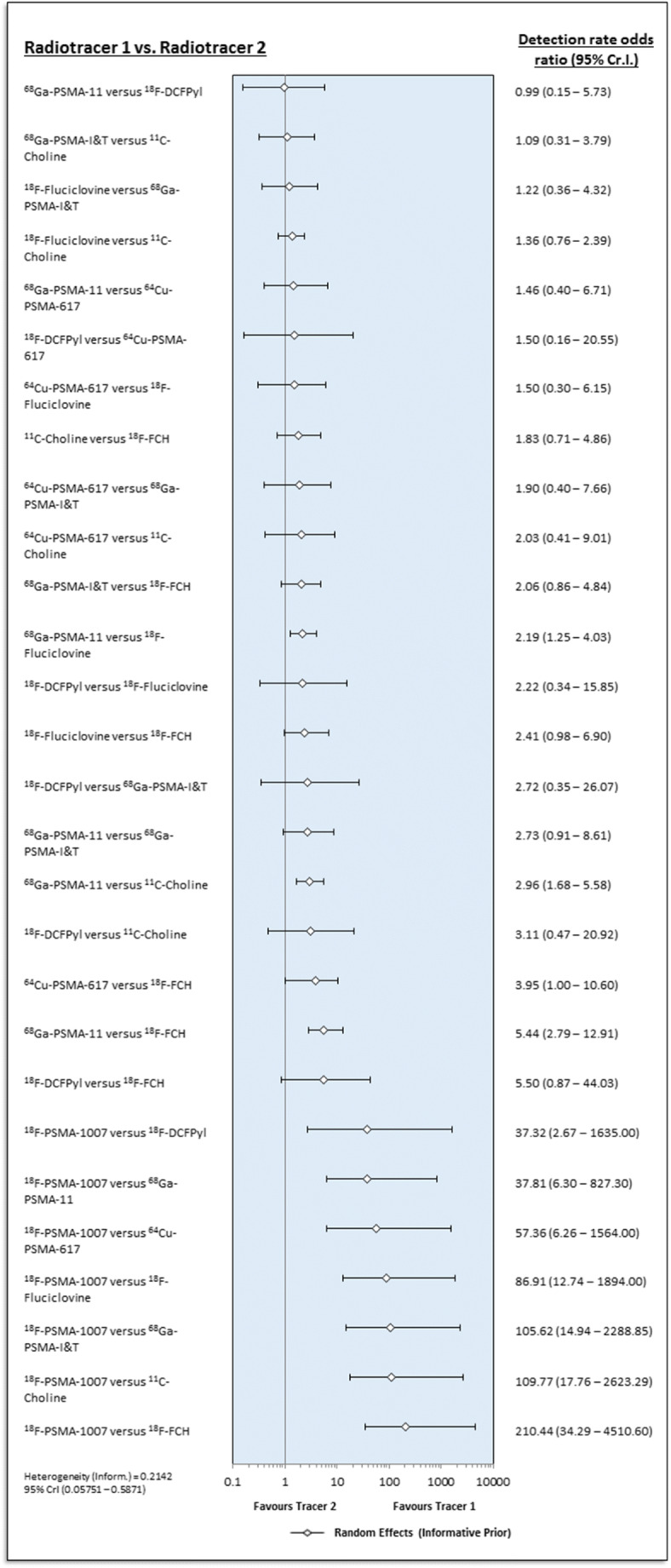
Forest plot comparing different radiotracers, including inferred comparisons from the network (random effects with informative priors)

The original article has been corrected.

