# Supplementary material for: Comparing the diagnostic performance of radiotracers in recurrent prostate cancer: a systematic review and network meta-analysis
Source: Eur J Nucl Med Mol Imaging. 2021 Feb 6;48(9):2978–89. doi: 10.1007/s00259-021-05210-9 (PMC8263438; doi:10.1007/s00259-021-05210-9)
Supplement: Supplementary file 1 — (DOCX 96 kb) [file 259_2021_5210_MOESM1_ESM.docx]

**SUPPLEMENTARY MATERIALS**

Boolean Search String:

(((PSMA-11) OR (PSMA-I&T) OR (THP-PSMA) OR (PSMA-HBEDD)) AND (PSMA-1007)) OR (((PSMA-11) OR (PSMA-I&T) OR (THP-PSMA) OR (PSMA-HBEDD)) AND (DCPFyl)) OR (((PSMA-11) OR (PSMA-I&T) OR (THP-PSMA) OR (PSMA-HBEDD)) AND (JK-PSMA-7)) OR (((PSMA-11) OR (PSMA-I&T) OR (THP-PSMA) OR (PSMA-HBEDD)) AND ((Fluclovine) OR (Fluorocholine) OR (FECH) OR (Fluciclovine) OR (FABC) OR (Choline) OR (Choline))) OR (((PSMA-11) OR (PSMA-I&T) OR (THP-PSMA) OR (PSMA-HBEDD)) AND (Choline)) OR (((PSMA-11) OR (PSMA-I&T) OR (THP-PSMA) OR (PSMA-HBEDD)) AND (RM2)) OR ((PSMA-1007) AND (DCPFyl)) OR ((PSMA-1007) AND (JK-PSMA-7)) OR ((PSMA-1007) AND ((Fluclovine) OR (Fluorocholine) OR (FECH) OR (Fluciclovine) OR (FABC) OR (Choline) OR (Choline))) OR ((PSMA-1007) AND (Choline)) OR ((PSMA-1007) AND (RM2)) OR ((DCPFyl) AND (JK-PSMA-7)) OR ((DCPFyl) AND ((Fluclovine) OR (Fluorocholine) OR (FECH) OR (Fluciclovine) OR (FABC) OR (Choline) OR (Choline))) OR ((DCPFyl) AND (Choline)) OR ((DCPFyl) AND (RM2)) OR ((JK-PSMA-7) AND ((Fluclovine) OR (Fluorocholine) OR (FECH) OR (Fluciclovine) OR (FABC) OR (Choline) OR (Choline))) OR ((JK-PSMA-7) AND ((Fluclovine) OR (Choline)) OR ((JK-PSMA-7) AND (RM2)) OR (((Fluclovine) OR (Fluorocholine) OR (FECH) OR (Fluciclovine) OR (FABC) OR (Choline)) AND (RM2)) OR (((Fluclovine) OR (Fluorocholine) OR (FECH) OR (Fluciclovine) OR (FABC) OR (Choline)) and (Choline)) OR ((Choline)) AND (RM2))

QUADAS-2 tool:

|  | Risk Of BIAS | | | | Risk of APPLICABILITY | | |
| --- | --- | --- | --- | --- | --- | --- | --- |
| **Study** | **PATIENT SELECTION** | **INDEX TEST** | **REFERENCE STANDARD** | **FLOW AND TIMING** | **PATIENT SELECTION** | **INDEX TEST** | **REFERENCE STANDARD** |
| Cantiello 2018 | Unclear | High | High | Unclear | Low | Low | Low |
| Dietlein 2015 | High | High | High | Unclear | Low | Low | Low |
| Pernthaler 2019 | Unclear | Unclear | Unclear | Unclear | Low | Low | Low |
| Witkowska 2019 | Unclear | High | High | Unclear | Low | Low | Low |
| Morigi 2015 | Unclear | High | High | Unclear | Low | Low | Low |
| Afshar-Oromieh 2014 | High | Low | Unclear | Unclear | Low | Low | Low |
| Calais 2019 | Low | Low | Low | Low | Low | Low | Low |
| Schwenck 2017 | Unclear | High | High | Unclear | Low | Low | Low |
| Emmett 2019 | Low | Low | Low | Low | Low | Low | Low |
| Nanni 2015 | Low | Low | Low | Unclear | Low | Low | Low |
| Nanni 2016 | Low | Low | Low | Unclear | Low | Low | Low |
| Bluemel 2017 | Unclear | High | High | High | Unclear | Unclear | Low |

Table S1: Risk of bias by domain as judged according to QUADAS-2 criteria.

| **Radiotracer** | **SUCRA** |
| --- | --- |
| **18F-PSMA-1007** | 0.9997 |
| **68Ga-PSMA-11** | 0.7385 |
| **18F-DCFPyl** | 0.6607 |
| **64Cu-PSMA-617** | 0.5626 |
| **18F-Fluciclovine** | 0.4242 |
| **68Ga-PSMA-I&T** | 0.3303 |
| **11C-Choline** | 0.2518 |
| **18F-FCH** | 0.03219 |

Table S2: SUCRA values

Convergence for the random effects informative model:

Fig S1: Convergence for the random effects (informative) model using the Brooks-Gelman-Rubin method.


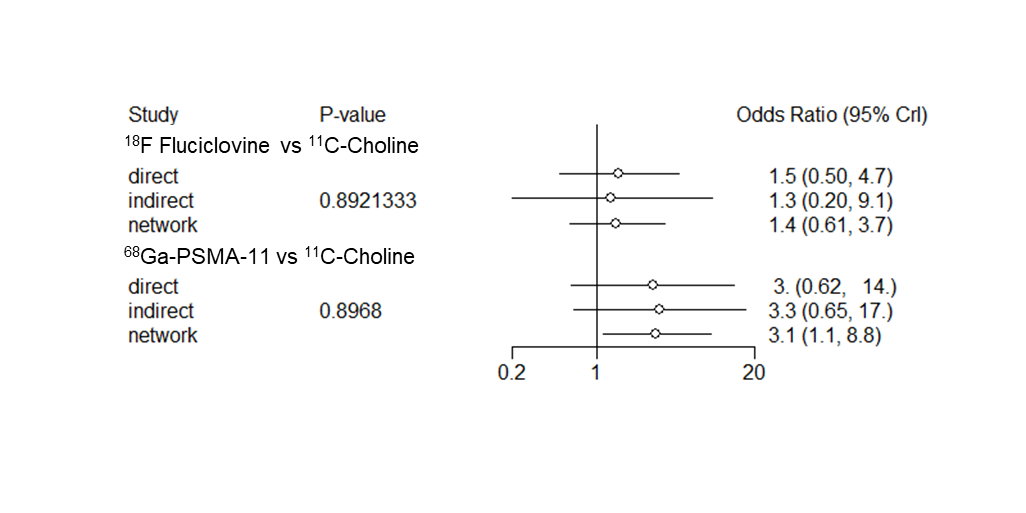


Fig S2: Split forest node. Using a closed loop within the network, direct and indirect evidence can be compared as a means of assessing confidence in the network results. Comparing results from the NMA with and without the direct evidence for each of the edges reveals no evidence of statistical inconsistency in any of the investigated contrasts
